# Comprehensive 4D velocity mapping of the heart and great vessels by cardiovascular magnetic resonance

**DOI:** 10.1186/1532-429X-13-7

**Published:** 2011-01-14

**Authors:** Michael Markl, Philip J Kilner, Tino Ebbers

**Affiliations:** 1 Department of Radiology, Medical Physics, University Hospital Freiburg, Germany; 2 CMR Unit, Royal Brompton Hospital and Imperial College, London, UK; 3 Center for Medical Image Science and Visualization, Linköping University, Sweden

## Abstract

**Background:**

Phase contrast cardiovascular magnetic resonance (CMR) is able to measure all three directional components of the velocities of blood flow relative to the three spatial dimensions and the time course of the heart cycle. In this article, methods used for the acquisition, visualization, and quantification of such datasets are reviewed and illustrated.

**Methods:**

Currently, the acquisition of 3D cine (4D) phase contrast velocity data, synchronized relative to both cardiac and respiratory movements takes about ten minutes or more, even when using parallel imaging and optimized pulse sequence design. The large resulting datasets need appropriate post processing for the visualization of multidirectional flow, for example as vector fields, pathlines or streamlines, or for retrospective volumetric quantification.

**Applications:**

Multidirectional velocity acquisitions have provided 3D visualization of large scale flow features of the healthy heart and great vessels, and have shown altered patterns of flow in abnormal chambers and vessels. Clinically relevant examples include retrograde streams in atheromatous descending aortas as potential thrombo-embolic pathways in patients with cryptogenic stroke and marked variations of flow visualized in common aortic pathologies. Compared to standard clinical tools, 4D velocity mapping offers the potential for retrospective quantification of flow and other hemodynamic parameters.

**Conclusions:**

Multidirectional, 3D cine velocity acquisitions are contributing to the understanding of normal and pathologically altered blood flow features. Although more rapid and user-friendly strategies for acquisition and analysis may be needed before 4D velocity acquisitions come to be adopted in routine clinical CMR, their capacity to measure multidirectional flows throughout a study volume has contributed novel insights into cardiovascular fluid dynamics in health and disease.

## Introduction

Phase contrast (PC) cardiovascular magnetic resonance (CMR) can measure, non-invasively, all three directional components of the velocities of blood flow relative to all four spatio-temporal dimensions of the heart and great vessels. The underlying principles have been known and applied over several decades [1-6]. The mapping of just the component of time-resolved velocity directed perpendicularly through a 2D plane is widely used for clinical measurements of volume flow [7-10]. This approach allows measurements of forward, regurgitant and shunt flows in congenital and acquired heart disease [11-14], and in certain circumstances, measurements of jet velocity. However, such acquisitions require appropriate placement of the velocity mapping plane, and clearly have limitations relative to the multiple directions of flow through the heart and large vessels [15].

Alternatively, Doppler ultrasound can be employed to assess regional blood flow velocities. The technique is widely and routinely used in numerous applications and cardiovascular pathologies and has a number of advantages compared to MRI including widespread availability, ease of use, and no contraindications in case of pacemakers or metallic implants. However, Doppler ultrasound is also limited by its inter-observer variability and by detecting only the component of velocity directed to or from the transducer.

Computed tomography provides relatively rapid 3D scans with excellent spatial resolution that can show the intravascular distribution of contrast agent at a given moment, but without being able to measure the velocities of blood flow.

This review paper deals principally with the methods and applications of the more comprehensive, 3 dimensional, 3 directionally encoded, time resolved (cine) velocity acquisition[16,17], which we will refer to as 4D velocity acquisition.

The concise naming of such acquisitions has varied between groups. While '4D' has come to be widely used and recognized, having the advantage of brevity, use of '3D cine' is arguably more correct and consistent with the use of '2D cine' to describe routine velocity mapping (see table 1 for a definition of terminology). It is important to be aware that the time dimension of this type of cine velocity acquisitions does not represent real time, but rather the time course of an effectively averaged heart cycle. Any instabilities or beat-to-beat variations of blood flow are not represented, as data contributing to each phase is gathered, by ECG triggering, from many heart cycles.

**Table 1:** Terminology regarding the dimensions and components of CMR velocity acquisitions.

Data acquisition method	Abbreviated description	Comment
3 dimensional, 3 directionally encoded, cine velocity acquisition	3D cine phase contrast, 3D cine velocity acquisition (more explicit) or 4D velocity acquisition (conveniently concise)	This is 'comprehensive' in the sense that all 3 directional components of velocity are measured with respect to all 3 spatial dimensions and the time course over the cardiac cycle.

2 dimensional, 1 directionally encoded (usually through-plane), cine velocity acquisition	2D cine phase contrast, 2D cine velocity mapping	Allows calculations of volume flow through a plane that transects a vessel.

As there is a risk of ambiguity, for example by taking '3D' to mean '3 directionally encoded' rather '3 dimensional', we recommend that the fuller description (left hand column) is included in the abstract and methods section of any relevant publication. Variations are possible, such as 2 dimensional and 3 directionally encoded cine velocity acquisitions, which would need to be identified explicitly. It would be incorrect to use '4D' and confusing to use '3D' in such a case.

Recent methodological advances including improved respiratory navigation, parallel imaging, or efficient radial k-space sampling allow good 4D velocity acquisition quality in acceptable time periods [18-20]. In combination with advanced flow visualization and quantification software, partially adapted from automotive and aerospace engineering, a tool for the studying multidirectional flow characteristics in the individual patients has been established [21-24].

In this article, the CMR methods used for 4D velocity acquisition, and the subsequent visualization and quantification of blood flow are reviewed. The value and limitations of this approach are considered in relation to a number of proposed clinical applications. We will focus on studies of blood flow in the thorax, although multidirectional velocity acquisitions can also be applied in other parts of the body such as the neck [25,26], brain [27,28] or liver [29], and, using a suitably low velocity encoding range, to studies of ventricular function [30-34].

## Methods

### 4D Velocity CMR Imaging

In 4D velocity acquisition, the combination of 3D spatial encoding, three-directional velocity encoding and cine acquisition provides data for the measurement and visualization of the temporal evolution of complex flow patterns throughout a 3D-volume. The acquisition of these very large datasets takes time and relies on efficient synchronization relative to cardiac and respiratory movements [16,18-20,35].

#### Pulse Sequence

**Figure 1:**
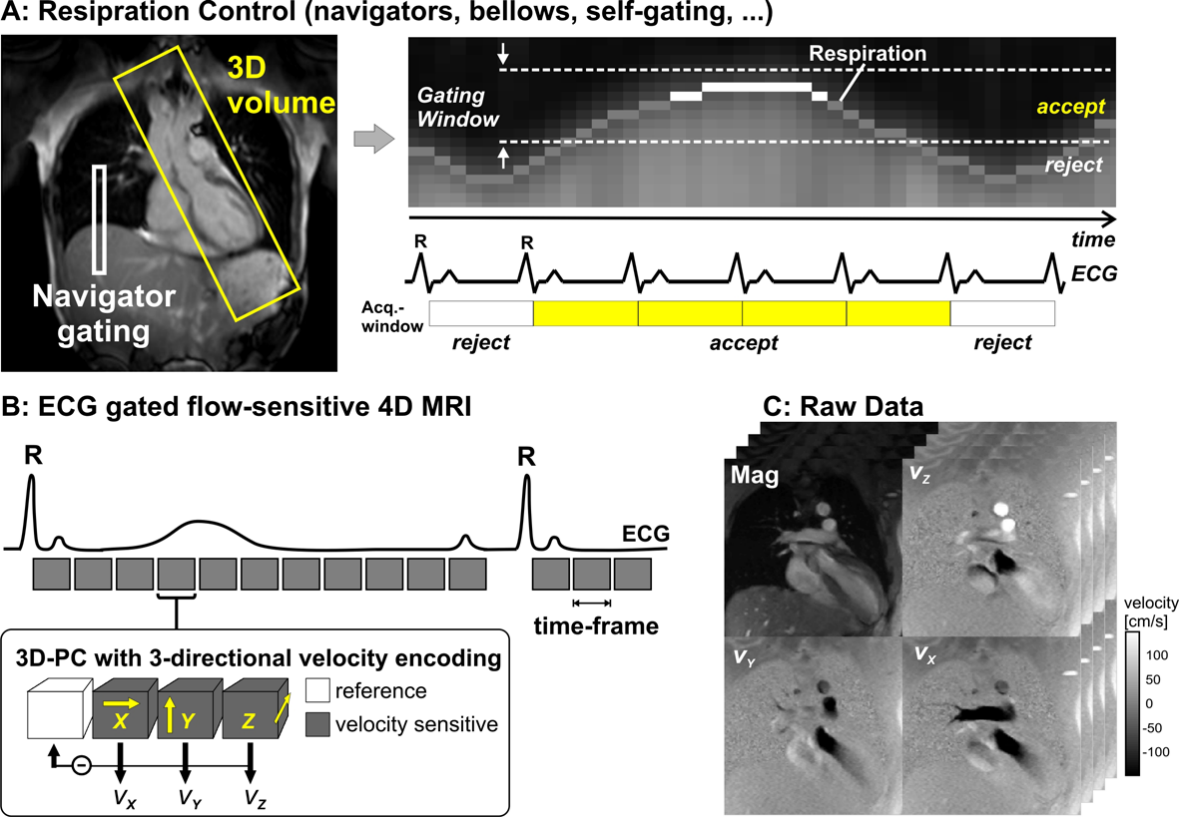
**Data acquisition for 3D cine velocity acquisition using navigator gating and prospective ECG gating**. Note that navigator control can also be replaced by other techniques for respiration estimation such as bellows or self-gating approaches. For each time-frame, flow compensated reference data and three velocity sensitive scans are acquired in an interleaved manner. Measurements are synchronized with the ECG cycle. The resulting raw data comprise information along all 3 spatial dimension, 3 velocity directions and time in the cardiac cycle.

A typical measurement strategy is schematically illustrated in figure 1. Data acquisition is based on rf-spoiled gradient echo sequences with short echo and repetition times of the order of TE = 2-4 ms and TR = 5-7 ms. The ECG signal is used to gate measurements to capture a series of time frames in the cardiac cycle for each R-R interval. During each cardiac cycle only a subset (n_k_) of all required (N) phase encoding steps are collected (k-space segmentation). The procedure has to be repeated for a total of N/n_k _heart beats to acquire the full data set. Gating can be accomplished using either prospective or retrospective gating. In prospective gating full 3D datasets are acquired at defined time points from the ECG trigger. In retrospective gating, full 3D datasets are retrospectively assembled by interpolation at percentages of the cardiac cycle. Prospective gating will be sufficient for studying systolic or early diastolic events, but differences in the length of involved heart cycles will prevent measurement at late diastolic phases. Using retrospective gating, full coverage of the cardiac cycle can be obtained.

Four acquisitions are necessary to complete the velocity encoding for each cine time frame. Successive acquisitions have to be performed for reference scan and three velocity sensitized acquisitions to calculate the three-directional blood flow velocity (v_x_, v_y_, v_z_). To minimize artifacts in phase difference images related to subject motion, interleaved velocity encoding is often performed, for which the different flow encodes are kept as close together as possible in time as shown in figure 1 (lower left). As a result, the highest achievable temporal resolution for this encoding strategy is T_Res _= 4 TR.

#### Acquisition Relative to Respiratory Movement

For the thoracic applications discussed here, respiratory motion during acquisition can cause severe artifacts. It remains impossible to acquire 4D velocity data within a breath hold, so effective and time-efficient respiratory gating is needed. Different approaches for monitoring respiratory position been proposed including bellows[36], CMR navigators [37-39], and self gating techniques [19,40].

Navigator respiratory gating is typically based on a measure of the diaphragm position using pencil beam or crossed pair navigators. Navigator measurements are interspersed with the ECG gating used to synchronizing data acquisition relative to the cardiac cycle. As an alternative, self gating techniques can be combined with data acquisition by adding a short data readout to the pulse sequences or by using alternative k-space sampling techniques such as radial data acquisition. The additional data can be analyzed in real time to detect respiration induced changes in signal intensity and phase.

For each method, the information on the current respiratory position is directly integrated into the data acquisition process in a near real-time manner. A gating window is defined relative to the end-expiratory position, and data is accepted when the breathing position falls within a predefined window. A certain amount of data is rejected; typically 30-60%, depending on the subject's breathing pattern and the width of the acceptance window. Adaptive k-space reordering, i.e. phase encoding based on the current position in the respiration cycle, can improve scan efficiency to 60-80% and so reduce overall scan time [18,41,42].

The combination of a number of recent methodological improvements (parallel imaging, respiration control with increased efficiency, time-optimized velocity encoding gradients, etc.) currently allows the acquisition of 4D velocity data with a spatial and temporal resolution on the order of 2 mm^3 ^and 40 ms, respectively, within reasonable scan times of the order of 10-20 minutes for a volume covering the thoracic aorta or 15-30 min to cover the heart [18,20,43,44]. The total scan time will depend on the heart rate and efficiency of respiration control in the individual patient. There can be flexibility regarding the selection of imaging parameters, with trade-offs between spatial resolution, temporal resolution and total scan time. Table 2 summarizes different imaging scenarios for a given cardiac period of *T*_*ECG *_= 800 ms.

**Table 2:** 4D PC-MRI imaging scenarios for a cardiac period of *T*_*ECG *_= 800 ms. PI = parallel imaging, R_net _= net acceleration factor

	venc [cm/s]	TR [ms]	3D volume [mm^3^]	matrix N_x _× N_y _× N_z_	No of phase encodes N = N_y _× N_z_	k-space segmentation n_k_	respiration efficiency R_eff _[%]	PI R_net_	spatial resolution [mm^3^]	temp. resolution T_Res _= 4 N/n_k _[ms]	total scan time N/n_k _*T_ECG_/R_net_/R_eff _[min]
Aorta	150	5.1	320 × 260 × 70	192 × 120 × 26	3120	2	70-80	1.8	1.7 × 2.2 × 2.5	40.8	14.4 - 16.5
	150	5.1	320 × 260 × 70	192 × 128 × 32	4096	2	70-80	1.8	1.7 × 2.0 × 2.2	40.8	18.9 - 21.7
	150	5.1	320 × 260 × 70	192 × 140 × 32	4480	3	70-80	1.8	1.7 × 2.0 × 2.2	61.2	12.6 - 14.5

Heart	100	5.8	290 × 290 × 108	96 × 96 × 36	3456	2	70-80	2	3.0 × 3.0 × 3.0	46.4	14.4 - 16.4
	100	5.8	290 × 290 × 100	116 × 116 × 40	4640	2	70-80	2	2.5 × 2.5 × 2.5	46.4	19.3 - 22.1
	100	5.8	290 × 290 × 108	96 × 96 × 36	3456	3	70-80	2	3.0 × 3.0 × 3.0	69.6	9.6 - 10.9

### Pre-Processing

It is well known that phase contrast CMR can be subject to phase offset errors associated with eddy currents, Maxwell terms, and gradient field imperfections [45-47]. These inaccuracies tend to increase with distance from the iso-center of the magnet. In traditional 2D velocity mapping, these errors can be limited by measuring flow in vessel segments at or near the iso-center of the magnet. For 4D acquisitions with large anatomic coverage, however, even small systematic inaccuracies can propagate into larger visualization errors with increasing distance from the iso-center. Although these distortions are well understood, correction methods may be absent or only partly applied. Appropriate corrections for these sources of error and consistent quality control of the data are important to ensure accurate 3D visualization and flow quantification. Post acquisition correction using a stationary phantom might be considered, but would require as much magnet time as the original acquisition.

Additional problems, such as velocity aliasing should be corrected in order to improve the data quality before further data processing or visualization. Appropriate correction and pre-processing strategies should thus be applied to ensure accurate flow measurement as described in more detail in references [46-48].

The exact correction approaches may need to be adapted to the specific CMR system, imaging protocol and cardiovascular region of interest. A systematic validation strategy, incorporating both in-vitro and in-vivo validation methods, is essential to assess whether the applied correction is effective for the chosen application [43,49].

### Phase Contrast Magnetic Resonance Angiography

**Figure 2:**
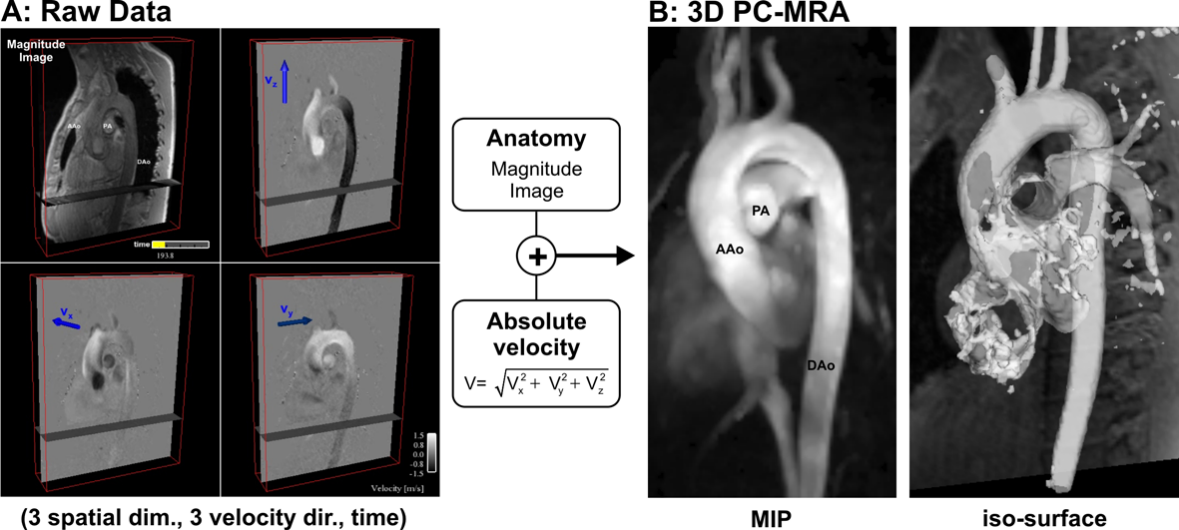
**Calculation of 3D PC-MRA for the thoracic aorta of a normal volunteer**. A 3D cine velocity acquisition (A) is used to calculate absolute velocities |v| for each image voxel which are additionally weighted by the magnitude images for suppression of background signal. **B**: The resulting 3D angiogram can be displayed as maximum intensity projection (MIP) or as a transparent 3D iso-surface which can be combined with 3D flow visualization as shown in figures 3 and 4. (AAo: ascending aorta, DAo: descending aorta, PA: pulmonary artery)

Three directional phase contrast data can not only be used for flow velocity analysis but also to gain information on vascular geometry by calculating a 3D phase contrast angiogram (3D PC MRA), information in the acquisition being used to identify vascular boundaries without the need for additional measurements [50-53]. Algorithms based either on absolute velocities combined with magnitude weighting, or on calculations of complex difference images have been proposed. An example is shown in Figure 2. Magnitude and three-directional velocities can be used to generate time-averaged 3D PC MRA by calculating absolute velocity images with background suppression by magnitude weighting. Further processing of 3D PC MRA data can be used to produce maximum intensity projection (MIP) or semi-transparent 3D iso-surface representations of the vascular geometry [20,54].

**Figure 3:**
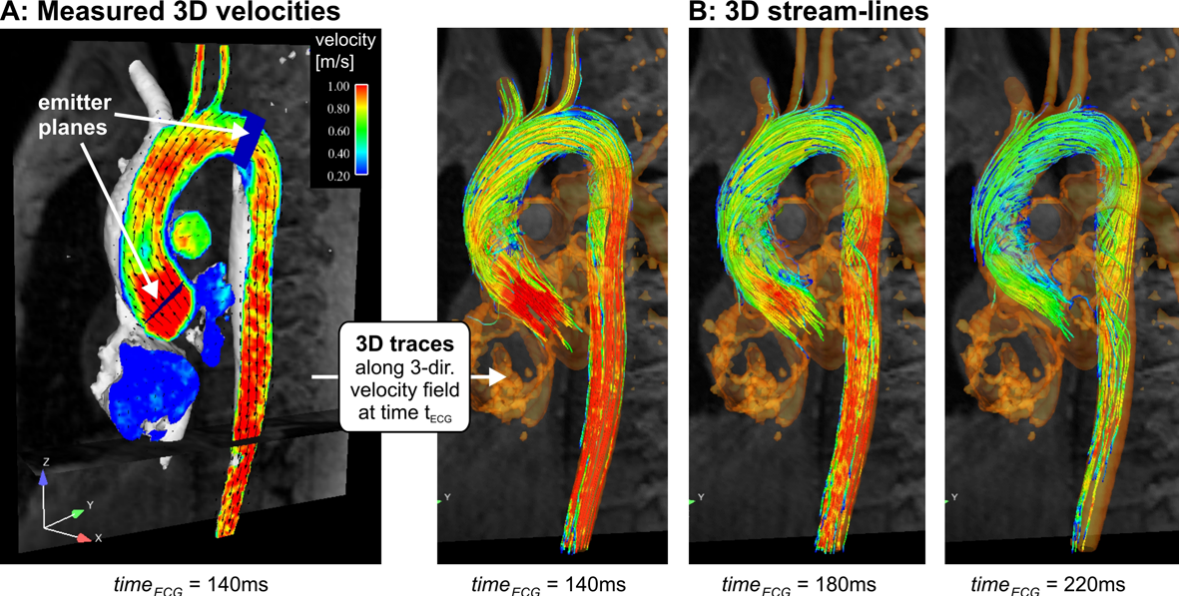
**3D visualization of normal peak systolic aortic blood flow using vector fields and color coding (A) and a series of systolic 3D streamlines (B)**. The gray shaded iso-surface represents the vessel lumen boundaries defined by the 3D PC-MR angiogram. Color coding reflects the local absolute velocity. Typical normal flow patterns such as flow acceleration near the aortic valve (A) or mild right-handed helical systolic flow in the ascending aorta (B) can be appreciated. See also additional file 1.

**Figure 4:**
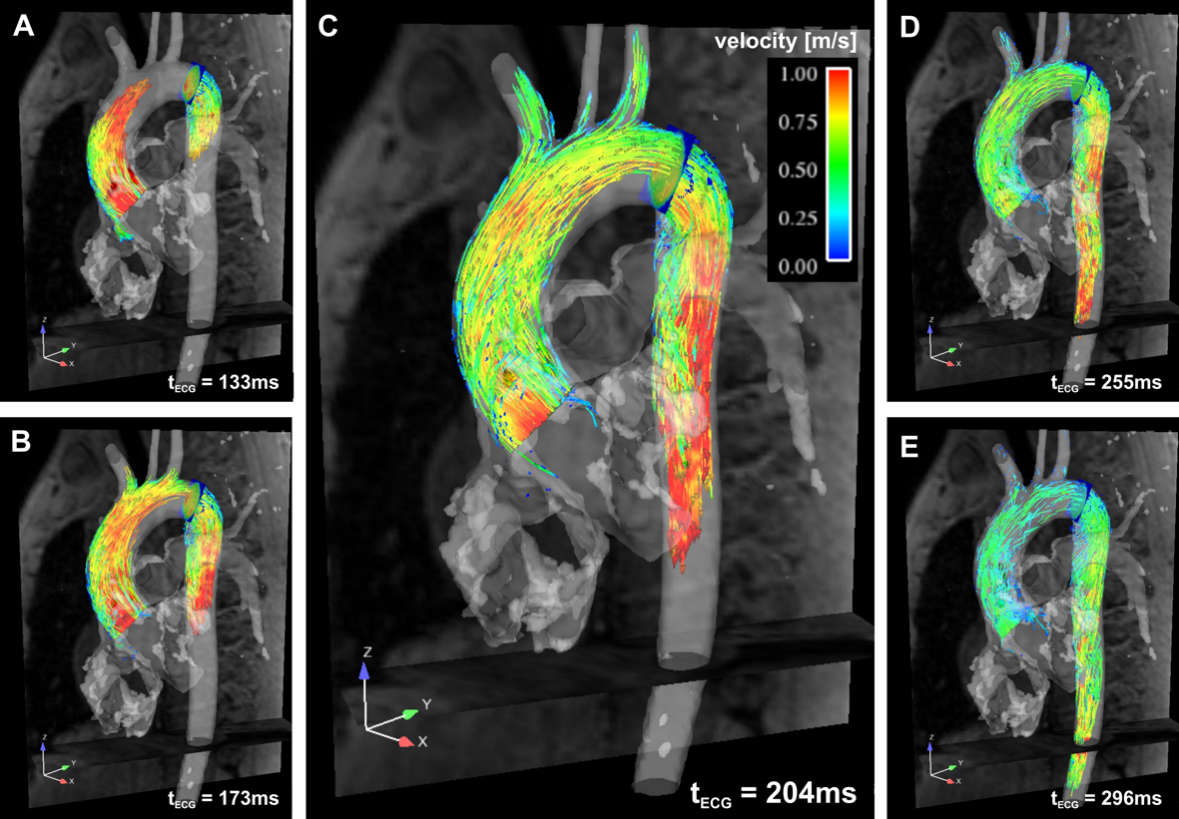
**3D pathlines in a normal thoracic aorta illustrating the temporal evolution of blood flow at five different instants in systole**. Pathlines were repetitively emitted at successive instants and originate from two emitter planes, one in the ascending aorta and one in the proximal descending aorta. Color coding reflects the local absolute velocity. See also additional file 2.

Although the derived 3D PC-MRA does not provide as detailed a depiction of vascular boundaries as contrast enhanced MRA, it can contribute to the presentation of flow results by displaying the 3D vascular form relative to which flows can be traced and orientated, as illustrated for the aorta in Figures 3 and 4.

**Figure 5:**
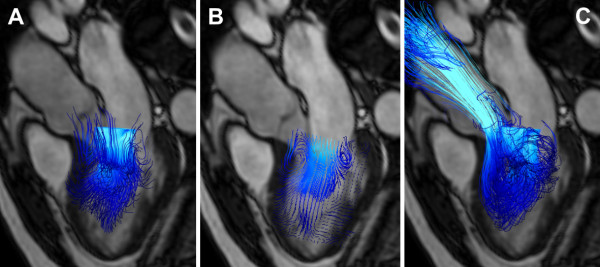
**Demonstration of different particle trace visualization techniques of early diastolic left ventricular inflow in a healthy volunteer**. **A**: Instantaneous streamlines traced at peak early diastolic inflow from a 20 × 20 plane across the mitral valve are used to visualize the directions of blood flow at this time point. **B**: short streamlines are here traced from multiple points on a 20 × 30 emitter plane oriented in the long axis of the left ventricle. **C**: Pathlines of virtual particles are traced from their positions at peak early diastolic inflow from a 20 × 20 emitter plane in the mitral valve and computed until end-systole, visualizing the flow paths though the left ventricle and into the ascending aorta. All particle traces are colored coded by velocity; blue represents low velocity, while turquoise represents higher velocity. A separately acquired balanced steady-state free precession three-chamber image provides anatomical orientation.

**Figure 6:**
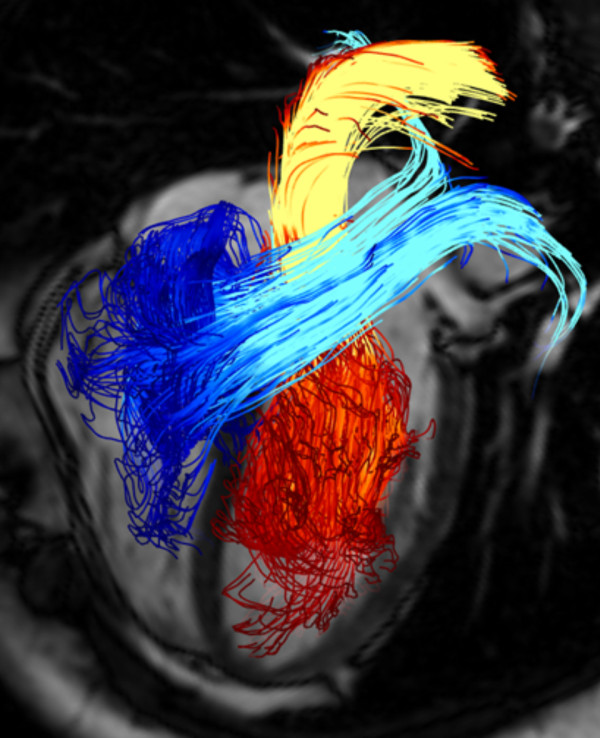
**Pathline visualization of cardiac blood flow**. Pathlines are traced from planes located at the mitral valve (red-yellow) and the tricuspid valve (blue-turquoise) at early diastolic ventricular inflow. A separately acquired balanced steady-state free precession three-chamber image provides anatomical orientation. See also additional file 3.

In the heart, the boundaries move considerably through the cardiac cycle, which makes 3D PC-MRA data less useful. Combining the flow visualization with separately acquired balanced steady-state free precession data, often part of the standard protocol, can improve orientation and presentation considerably, as demonstrated in Figures 5 and 6.

### Data Analysis

The comprehensive nature of the 4D velocity data (3 spatial dimensions, 3 directional velocity encoding, and time) requires data analysis strategies to deliver clinically relevant visualization and quantification. A typical CMR exam of the thoracic aorta comprises about 4000-8000 raw magnitude and velocity component images, depending on the spatial and temporal resolution and coverage. Efficient visualization and quantification strategies are needed to convert the wealth of information contained into meaningful images and measurements. Interactive methods of analysis offer versatility for the exploration of novel or alternative approaches, whereas standardized and automated methods can be more user-friendly, less operator dependent, and so more suitable for clinical use or larger patient studies.

#### Visualization - Vector Fields, Streamlines & Pathlines

For the analysis and visualization of multidirectional blood flow in a 3D volume, several visualization tools, including 2D vector-fields, 3D streamlines and 3D pathlines (Table 3) have been reported [21-24,55-58]. It should be said at the outset that it is not possible to depict clearly, without overlap, all of the velocities measured in a volume of pulsatile, multidirectional flow, even with animation on computer screen, let alone by means of static images on a printed page. Choices of visualization methods need to be made, potentially offering different and complementary representations of the same flow field.

**Table 3:** Explanations of terms relating to CMR velocity acquisition, blood flow visualization, flow and wall properties.

Dimension	Dimension	A dimension refers, in this paper, to one of up to 3 orthogonal (X, Y, Z) spatial dimensions and the temporal dimension, along each of which a series of measurements of velocity components are made. For practical purposes, the spatial dimensions are subdivided into voxels and the time dimension, into a number of phases of an effectively averaged cardiac cycle.
	
	Directional component	One of up to three orthogonal (X, Y, Z) components of a vector such as velocity, each usually represented by a single number at any given point in space-time.
**CMR methods**	Phase contrast velocity acquisition	The encoding of velocity in CMR by means of differences of phase between MR signals recovered using two differently velocity encoded acquisition sequences, applied one after the other, usually in quick succession.
	
	Phase contrast Intravoxel velocity standard deviation	Quantification of the standard deviation of the blood flow velocity distribution within a voxel obtained from the magnitude of phase contrast MRI signals acquired with different first gradient moments. Intravoxel velocity standard deviation can be used to derive turbulence intensity.
	
	Fourier velocity encoding	This technique entails a measurement of the spectrum of one or more components of velocity reflecting a 'dimension'. These could represent multiple velocities that might be measured in a single, spatially or temporarily extended flow region in an unstable flow field.

**Visualization**	Vector plot	A line or arrow representing both the magnitude and direction of velocity at a point, calculated from the magnitudes of X, Y and Z components of velocity, or X and Y if in a single plane.
	
	Particle trace	The computed behavior of a virtual particle in a flow field. In pulsatile flow several types of particle traces can be computed, as instantaneous streamlines and pathlines
	
	Streamline	A line plotted through in a flow field at a given instant in time such that it is aligned with the local velocity vectors at all points along its length.
	
	Instantaneous streamline	A streamline plotted at a specific instant in a changing flow field.
	
	Pathline	A line through a flow field representing the path traced by a particle or virtual particle released from a given seed point in the flow field.

**Flow features and properties**	Viscosity	A measure of the resistance of a fluid to internal deformation.
	
	Vortex	Rotating or swirling motion in a flow field, where streamlines or pathlines tend to curl back on themselves
	
	Helical flow	Part of a flow field with rotation around an axis of flow such that streamlines are helical
	
	Unstable flow or flow instability	Irregularly fluctuating disturbances of flow consisting of multiple eddies and counter-eddies. Several factors in the heart and great vessels predispose to instability: relatively large luminal diameters, sudden changes of lumen diameter, relatively high flow velocities, velocity changes (particularly deceleration), flow separation and shear within the flow field.
	
	Turbulence	A fluid regime characterized by randomly and rapidly fluctuating velocities.
	
	Flow separation	The separation of streamlines in a flow field from an adjacent wall. This tends to happen by virtue the forward momentum of flow relative to a curving, irregular or discontinuous boundary, for example where venous flows enter the atria, separate from the tips of valves or where streamlines separate from the inner curvature the distal aortic arch.
	
	Recirculation	The recirculation of streamlines or pathlines from a forward stream back into a separation zone, for example beyond each heart valve.
	
	Shear	Shear in a flow field is fluid deformation such that adjacent layers move relative one another, for example in the shear layer between a high velocity jet and the adjacent low velocity fluid. In CMR, high shear can results in a range of velocities in a single voxel which can cause local loss of blood signal due to dephasing.
	
	Pulse wave velocity	The velocity of propagation of a pulse wave along a vessel, usually an artery, normally several times faster than the velocities of blood flow within the vessel.
	
	Wall shear stress	Refers to the stress, meaning force per unit area, parallel to the wall, exerted by shear in the fluid layer immediately adjacent to the wall (fluid-wall shear stress).

**Wall**	Intramural stress	Intramural stress is a measure of the internal forces acting within a deforming vascular or cardiac wall. The intramural stress dependents on variables such as transmural pressure, wall curvature, wall thickness and any constraints from the outside. The resultant strains depend on the structural properties of the wall.

A streamline is instantaneously tangent to the velocity vector of the blood flow, while pathlines resemble paths of virtual particles released within the time-varying blood flow velocities. In a continuous, stable, non-pulsatile flow field, streamlines and pathlines would be identical. However, they differ from each other in pulsatile flows such as those in the heart and great vessels. In the cyclically changing velocity fields that can be measured by 4D velocity CMR, the shapes of instantaneous streamlines change from time frame to time frame. The appearance of pathlines will, on the other hand, depend on the time frame used for virtual particle emission.

Examples of visualization of aortic flow using streamlines and pathlines are shown in figures 3 and 4, respectively. In these, the previously calculated 3D PC angiogram (figure 2) is used to aid anatomical orientation, co-register vascular geometry and 3D blood flow, and position emitter planes for streamlines or pathlines.

Figure 3 illustrates flow visualization using streamlines in the entire thoracic aorta based on the underlying vector field. The measured peak systolic velocity vectors are shown as arrows with color representing local speed in a plane aligned with the aorta (figure 3A, and additional file 1). To appreciate the 3D distribution of flow at the same timeframe, emitter planes were positioned across the vessels of interest as shown in figure 3A, and streamlines were calculated forward and backward to give 3D traces that show the directions of instantaneous flow velocities at all points along their length. Streamlines represent the directions of flow at any chosen instant in time but do not show the paths that would be traced by actual moving blood cells over time.

Pathlines, as shown in figure 4 and additional file 2, are emitted at a chosen time-frame and incorporate velocity information through subsequent phases of the cardiac cycle. By repetitively emitting pathlines at successive instants, time-resolved pathlines can be plotted, depicting the traces of virtual particles through the flow field according to the measured speeds and directions of flow. The resulting traces reflect the dynamics of 3D blood flow over the cardiac cycle.

Clearly stating the used particle trace visualization technique is therefore crucial for the interpretation of static images.

Both streamlines and pathlines can be color coded according to the local blood flow velocity magnitude and spatially interpolated based on a chosen density of emitter points arrayed across the emitter planes (figures 3 and 4). Alternatively, streamlines and pathlines can be color coded according to their vascular origin to improve the understanding of flow paths for complex cardiovascular geometries as in congenital heart disease [59].

Streamlines can also be used to visualize the blood flow in the heart (Figure 5A). Spatial overlap and the marked changes of flow direction make visualization and interpretation more challenging in this region. The 2D emitter planes are not easy to place consistently relative to the heart chambers and they rarely allow the visualization of all chambers simultaneously. Here, a large number of short streamlines, released from a 2D plane aligned with the principal direction of flow, can give an overview of intracavity flow, as illustrated in figure 5B. Pathline visualization can provide appealing visualizations, showing the paths traced by blood through the chambers (see figure 5C, Figure 6 and additional file 3). Alternatively, pathlines can be emitted from a 3D volume, allowing for the tracking of this volume of blood forward or backward in time.

In addition to visualization, pathlines can be used as an internal check of data quality. A large number of pathlines inappropriately leaving the blood pool indicates insufficient data quality, which might be due to noise, background error, or insufficient spatial or temporal resolution [24,43].

**Figure 7:**
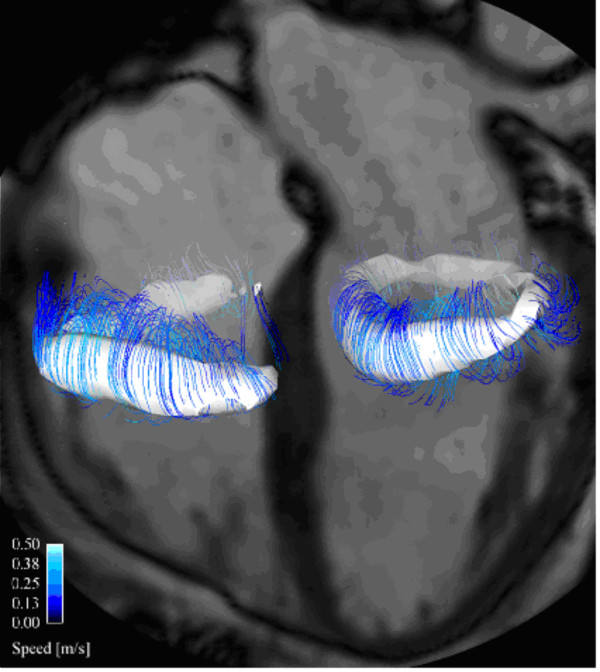
**Characterization of blood flow in the human heart of a healthy volunteer during late diastolic inflow**. Automatically detected vortex cores are shown as white isosurfaces and streamlines are traced around these isosurfaces to enhance the visualization. A (partial) vortex ring can be seen below the mitral valve (right in image) and the tricuspid valve (left in image). See also supplement additional file 4.

To make the analysis of these large velocity dataset less time-consuming or user-dependent, automatic extraction and visualization of flow structures of interest can be applied. The majority of these approaches have been tailored for computational fluid dynamic applications, which tend to deliver higher resolution and less noise. Flow characterization using vector pattern matching has been tested successfully on 4D velocity data [60]. Using this approach, flow structures such as vortical, helical, divergent, convergent or parallel flow can be detected automatically and used for quantification or visualization purposes, as shown in figure 7 and additional file 4.

### Quantification

Since 3D cine velocity data represents the underlying time-resolved velocity vector field, albeit effectively averaged over many cycles, it is possible to quantitatively evaluate regional flow and velocity parameters (total flow, flow rates, peak velocities, retrograde flow, etc.) [10-14,61]. Moreover, the complete spatial and temporal coverage and measurement of the three-directional velocity information permits the calculation of derived hemodynamic parameters such as pressure differences [62-64], wall shear stress [61,65,66], pulse wave velocity[67-70], turbulence intensity[71-73] and more.

#### Blood Flow and Shear Rates

**Figure 8:**
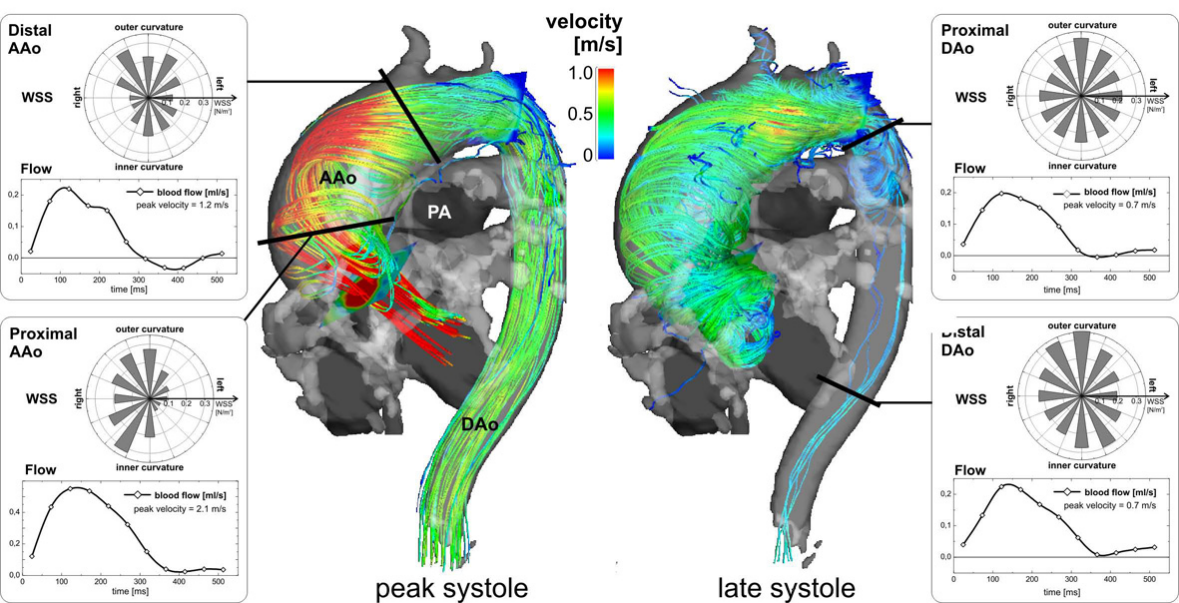
**Flow analysis in a patient with an ascending aortic aneurysm (maximum diameter = 51 mm)**. 3D stream lines clearly show asymmetric aortic outflow and an accelerated flow channel along the outer aortic curvature and the onset of substantial helical flow during peak systole. The flow helix grows until late systole to occupy the entire ascending aorta and arch. Retrospective quantitative analysis in 4 planes (black lines) was used to evaluate the impact of the altered flow patterns on flow and wall parameters. The complex flow resulted in considerable variation of peak velocities along the aorta. The segmental distribution of wall shear stress (WSS, polar plots) in the ascending aorta (AAo, left) showed heterogeneity along the aortic circumference reflecting the pronounced asymmetry of flow and helix formation. Low WSS at the inner and left aortic curvature or also abnormally high WSS may be associated with altered endothelial function and indicate vascular regions at risk for further arterial remodeling. In contrast, flow in the descending aorta (DAo, right) was relatively normal with more homogenous segmental WWS distribution, i.e. high and more constant WSS along the entire lumen circumference. To view the temporal evolution of 3D aortic blood flow by time-resolved 3D pathlines see additional file 5. (PA: pulmonary artery).

An advantage of 4D velocity acquisition relative to traditional 2D velocity mapping is its suitability for retrospective quantitative analysis at any anatomical location within the acquired volume. An example of a strategy for retrospective quantification is illustrated in figure 8 for a patient with a large ascending aortic aneurysm. Three dimensional flow visualization using systolic streamlines was combined with quantitative analysis in user selected planes across the ascending and descending aorta. Considerably altered flow patterns compared to the normal volunteer in figures 3 and 4 are evident (for pathline visualization see additional file 5). The three-directional velocities inside the vessel contours were used to calculate flow-time curves, peak systolic velocities, and segmental wall shear stress (WSS, blood shear rates near the vessel wall). The cumulative results shown in figure 8 illustrate the potential for analysis of several different parameters from a single 3D cine velocity acquisition (for details see figure 8 caption).

Advantages of 4D relative to the more conventional acquisitions of several, specifically located 2D velocity maps include simpler planning and retrospective choice of locations and types of analysis. Volumetric flow may be calculated in regions not previously known to be relevant, identified retrospectively and interactively. An example of such an approach is the measurement of flow through all four heart valves, using retrospective tracking of the valves [74]. 4D acquisition potentially enables quantification of forward and regurgitant flow volumes through all four valves and shunt flow, if present, from a single, albeit relatively prolonged acquisition.

#### Pathline Based Quantification in the Heart

**Figure 9:**
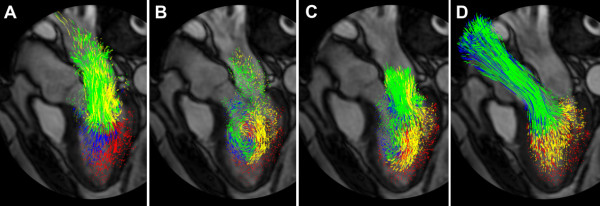
**Pathline visualization of blood flow during one cardiac cycle in the left ventricle (LV) of a healthy, 61 year old male at peak early LV filling (A), diastasis (B), peak atrial contraction (C), and peak systole (D)**. The pathlines are color coded to distinguish 4 different compartmental behaviors through the cardiac cycle: *Direct Flow *(green) enters the LV during diastole and leaves the LV during systole in the analyzed heart beat, *Retained Inflow *(yellow) enters the LV during diastole but does not leave during systole in the analyzed heart beat, *Delayed Ejection Flow *(blue) starts and resides inside the LV during diastole and leaves during systole, *Residual Volume *(red) resides in the LV through more than two cardiac cycles. See also additional file 6.

The forward and backward emission of pathlines from all parts of the left ventricular blood pool at the onset of systole has been used in analyses of intraventricular flow [43]. After identifying the short isovolumetric phase between mitral closure and aortic opening, automatic assessment of the flow patterns was implemented. The number of traces entering the ventricle should be equal the number leaving, providing a data driven measure of its quality for pathline visualization. This complements quality control performed by in-vitro experiments. Further, by assuming that every pathline represents the movement of the amount of blood defined by the emitter density, the cardiac output can be estimated by multiplying the number of traces passing the aortic valve with the emitter volume. This can be compared with other measurements of global cardiac function [43]. Using pathline based analysis, the intraventricular flow was subdivided into 4 components according to their rates of passage relative to the cycle[43,75,76]. *Direct Flow*: Blood that enters the LV during diastole and leaves the LV in the following systole. *Retained Inflow*: Blood that enters the LV during diastole but does not leave during systole in the analyzed heart beat. *Delayed Ejection Flow*: Blood that starts and resides inside the LV during diastole, but then leaves with the following systole. *Residual Volume*: Blood that stays in the LV for more than two cardiac cycles. This information can be used to color-encode blood regions based on their rates of passage, giving an informative type of visualization (as shown in figure 9 and additional file 6), and a quantitative measure of ventricular function in relation to blood transit [43,75].

#### Pressure Difference Mapping

The acquired velocity vector field with its boundaries identified can also be used for the calculation of the intra-compartmental differences of pressure that are associated with the momentum changes of blood. For laminar flow conditions, pressure gradients can be calculated directly from the Navier-Stokes equations, assuming blood to be an incompressible, Newtonian fluid [77]. This approach allows the estimation of temporally and spatially distributed gradients and differences of pressure across a large vessel segment or cardiac chamber. In principle, spatial integration of these gradients results in time-resolved 3D pressure difference maps. To circumvent problems with integration path dependency due to noise in the gradient data and truncation errors, the pressure Poisson equations (PPE) are normally solved [62-64] which, for laminar flow conditions, is thought to give reasonably accurate relative pressure fields [78].

#### Turbulence

**Figure 10:**
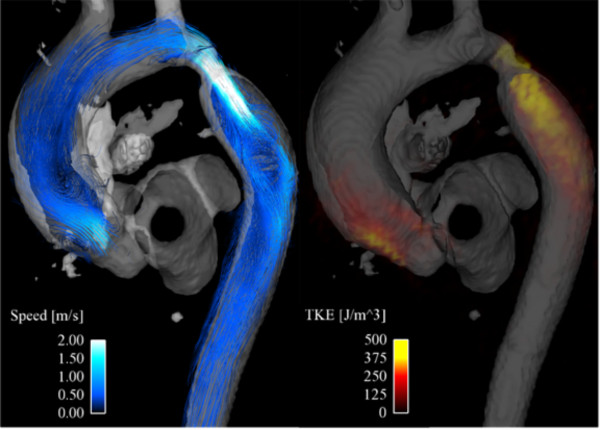
**Streamline visualization of blood flow (left) and volume rendering of turbulence intensity (turbulent kinetic energy, TKE) (right) at peak systole in a patient with an aortic coarctation distal to the left subclavian artery**. In addition to the coarctation, elevated values of turbulence intensity can be seen in the ascending aorta, resulting from a minimally obstructive sub-aortic valve membrane. A time-resolved volume rendering visualization of the TKE is provided in additional file 7.

Disturbed and turbulent blood flow, characterized by fast random temporal and spatial velocities fluctuations, appears to be present in many cardiovascular diseases. These irregular and rapid fluctuations are not represented in the mean intravoxel velocities calculated from a 4D velocity acquisition. However, the magnitude of the signal can be used as a measure of the standard deviation of the velocity distribution within the voxel [71,72], which is related to the turbulent kinetic energy (TKE), a direction independent measure of turbulence intensity [71]. The velocity and the turbulent kinetic energy combined can give a visualization of disturbed flow, as shown in figure 10 and additional file 7 for an aortic coarctation. The velocity field at peak systole is visualized using streamlines while the turbulence intensity is color coded according to the scale shown. Note also the elevated values of turbulent kinetic energy in the aortic valve caused by a minimally obstructive subvalvular membrane, showing the sensitivity of this approach to abnormally disturbed flow.

## Applications

Several studies have shown the potential of 4D velocity acquisition for detection of pathologically altered flow characteristics in the heart and adjacent large vessels, suggesting possible roles fluid dynamic factors in the initiation or progression of pathology. A summary of selected patient studies and their major findings is given in Table 4.

**Table 4:** Summary of selected studies using 3 directionally encoded CMR velocity acquisitions in the heart and vessels.

Anatomy	Study	Title	Subjects	Findings
**Heart**	Kim et al. * J Am Coll Cardiol 1995 [81]	Left ventricular blood flow patterns in normal subjects: a quantitative analysis by three-dimensional magnetic resonance velocity mapping	n = 26	Diastolic vortex formation in the left ventricle, in close temporal relation to the motion of the anterior mitral leaflet
	
	Kilner PJ et al. * Nature 2000 [79]	Asymmetric redirection of flow through the heart.	n = 22	Asymmetric redirection of streaming blood in atrial and ventricular cavities of the adult human heart
	
	Fyrenius A et al. Heart 2001 [56]	Three dimensional flow in the human left atrium.	n = 11	Global left atrial flow in the normal human heart comprises consistent patterns specific to the phase of the cardiac cycle
	
	Kozerke et al. J Magn Reson Imaging 2001 [23]	Visualization of Flow Patterns Distal to Aortic Valve Prostheses in Humans Using a Fast Approach for Cine 3D Velocity Mapping	n = 6	Distinct flow patterns reflecting aortic valve design were observed close to the valve. Further downstream, flow patterns varied considerably indicating the impact of varying aortic anatomy
	
	Bolger AF et al. J Cardiovasc Magn Reson 2007 [75]	Transit of blood flow through the human left ventricle mapped by cardiovascular magnetic resonance.	n = 18	Paths, compartmentalization and kinetic energy changes of blood flowing into the LV.
	
	Roes et al. Invest Radiol 2009 [91]	Flow assessment through four heart valves simultaneously using 3-dimensional 3-directional velocity-encoded magnetic resonance imaging with retrospective valve tracking in healthy volunteers and patients with valvular regurgitation	n = 29	Net flow volumes through the 4 heart valves were compared in 22 healthy volunteers and in 29 patients with ischemic cardiomyopathy who were suspected of valvular regurgitation.
	
	Eriksson et al. J Cardiovasc Magn Reson 2010 [43]	Semi-automatic quantification of 4D left ventricular blood flow	n = 9	Semi-automatic payhline analysis for the quantification of 4D blood flow resulted in accurate LV inflow and outflow volumes and high reproducibility

**Large vessels**	Kilner PJ et al. * Circulation 1993 [55]	Helical and retrograde secondary flow patterns in the aortic arch studied by three-directional MR velocity mapping.	n = 10	Helical and retrograde streams are consistent features of intra-aortic flow in healthy subjects
	
	Bogren HG et al. ** J Thorac Cardiovasc Surg 1995 [110]	Magnetic resonance velocity vector mapping of blood flow in thoracic aortic aneurysms and grafts.	n = 13	Altered flow patterns were found to be associated with altered vessel geometry
	
	Bogren HG et al. ** J Magn Reson Imaging 1997 [111]	Blood flow patterns in the thoracic aorta studied with three-directional MR velocity mapping: the effects of age and coronary artery disease.	n = 28	Significantly different flow characteristics in normal subjects compared with patients and during ageing
	
	Kvitting et al. J Thorac Cardiovasc Surg 2004 [86]	Flow patterns in the aortic root and the aorta studied with time-resolved, 3-dimensional, phase-contrast magnetic resonance imaging: Implications for aortic valve-sparing surgery	n = 8	Patients with Marfan syndrome 6 months after aortic valve-sparing surgery with straight Dacron grafts and normal volunteers
	
	Bogren HG et. Al ** J Magn Reson Imaging 2004[112]	4D MR velocity mapping of blood flow patterns in the aorta in patients with atherosclerotic coronary artery disease compared to age-matched normal subjects.	n = 41	Increased retrograde velocity in patients with atherosclerosis compared to normal subjects. The aging process has a similar effect on blood flow patterns as atherosclerosis.
	
	Markl M et al. J Thorac Cardiovasc Surg 2005 [85]	Time-resolved three-dimensional magnetic resonance velocity mapping of aortic flow in healthy volunteers and patients after valve-sparing aortic root replacement.	n = 22	Altered aortic flow dynamics in patients undergoing various types of valve-sparing aortic root replacement.
	
	Reiter G, et al Circ Cardiovasc Imaging 2008 [88]	MR-derived 3D blood flow patterns in the main pulmonary artery as a marker of pulmonary hypertension and a measure of elevated mean pulmonary arterial pressure.	n = 48	Vortices of blood flow in the main pulmonary artery enable the identification of manifest pulmonary hypertension. Elevated mean pulmonary arterial pressures is related to vortex duration
	
	Frydrychowicz A, et al. J Magn Reson Imaging 2009 [89]	Three-dimensional analysis of segmental wall shear stress in the aorta by flow-sensitive four-dimensional-MRI.	n = 31	Normal distribution of vectorial WSS and OSI in the entire thoracic aorta derived from flow-sensitive 4D-MRI data
	
	Harloff A et al. Magn Reson Med 2010 [96]	In vivo assessment of wall shear stress in the atherosclerotic aorta using flow-sensitive 4D MRI.	n = 93	Predictive value of WSS for plaque existence depends on the aortic segment. Locations of critical wall parameters move to neighboring segments of regions affected by atherosclerosis
	
	Hope MD et al. Radiology 2010 [58]	Bicuspid aortic valve: four-dimensional MR evaluation of ascending aortic systolic flow patterns.	n = 53	Abnormal helical systolic flow in the ascending aorta of patients with a bicuspid aortic valve.
	
	Markl M et al. Magn Reson Med 2010 [68]	Estimation of global aortic pulse wave velocity by flow-sensitive 4D MRI.	n = 46	Pulse wave velocity based on four-dimensional MRI data was higher in patients with atherosclerosis compared to age-matched controls and younger volunteers
	
	Hope MD et al. J Magn Reson Imaging 2010 [44]	Clinical evaluation of aortic coarctation with 4D flow MR imaging.	n = 34	Hemodynamic significance was established by evaluating collateral blood flow. Distorted blood flow patterns in the descending aorta were detected after coarctation repair
	
	Harloff A et al. Stroke; 2010 [93]	Complex plaques in the proximal descending aorta: an underestimated embolic source of stroke.	n = 94	Retrograde flow from complex plaques in the descending aorta can explain embolism to all brain territories as a new source of stroke.

Earlier studies acquired this data relative to 2D cine velocity acquisitions in oblique* or stacked** planes. The remainder used 3D cine (4D) acquisitions.

The following sections give an overview over the major findings and applications of 4D velocity acquisition for the assessment of the whole heart and adjacent large vessels.

### Understanding Blood Flow in the Healthy Cardiovascular System

#### Heart

Multidirectional velocity mapping in healthy volunteers contributed visual evidence in support of a hypothesis concerning the functional advantages of the direction changes and asymmetries of flow which follow from the looped curvatures of the heart [79]. The left atrium tends to be regarded as a conduit during diastole and a reservoir during systole. 4D velocity acquisitions revealed vortical flow during systole and diastole [56]. The blood redirected in the principal vortex originated mainly from the left pulmonary veins. Inflow from the right veins joined the vortex periphery, between the vortex and the atrial wall. These consistent patterns and their progression through the cycle may help to minimize both stasis and energy dissipation in a healthy subject in sinus rhythm.

During ventricular diastole, atrial blood passes down through the mitral valve to enter the left ventricle. In the resting heart, this occurs in an early diastolic phase as the ventricle relaxes and recoils, and in late diastole as the left atrium contracts. The ratio between early and late diastolic inflow is an important clinical parameter in the evaluation of diastolic function. Using 4D velocity data, it was shown that early and late diastolic filling have different directions and locations of the peak velocity, which has implications for evaluation of diastolic function by Doppler ultrasound [80].

Flow patterns within the ventricles tend to be dominated by the diastolic inflow with asymmetric, regionally constrained ring vortices beginning to develop beneath both mitral and tricuspid leaflets [79,81], as seen in figure 7 and additional file 4. The complex, mobile geometries of the ventricles and the two phases of resting inflow can generate additional vortices with variable size and position, which accelerate and decelerate over time [81]. Using pathline based analysis, it was found that in the normal heart about 1/3 of the left ventricular end-diastolic volume is *Direct Flow*, blood that enters the LV during diastole and leaves the LV during systole in the analyzed heart beat [43,75,76].

#### Large vessels

Several groups have reported the application of 4D velocity acquisition to the evaluation of normal flow characteristics and derived hemodynamic parameters in the large vessels (aorta and pulmonary artery) [15,22,44,55,82-84].

In the healthy thoracic aorta, characteristic flow patterns such as helical flow and mild early diastolic retrograde flow in the ascending aorta have been identified. Normal large scale aortic flow features include the following: During systole, left ventricular outflow initially generates flow that tends to be skewed towards the shorter, inner curvature. The peak velocity stream tends to migrate to the outer curvature, and later in systole, to curve postero-laterally, back towards the inner curvature in a right-handed helix in the ascending aorta and arch. Flow velocities are highest in the ascending aorta (up to about 150 cm/s in the slight vena contracta above the outflow tract and valve) and slightly lower in the arch, where flow enters the brachiocephalic branches. In the proximal descending aorta, velocities increase where streamlines tend to separate from the inner curvature, converging slightly towards the outer wall (see also color coding of streamlines and pathlines in figures 3 and 4). During early diastole, retrograde flow occurs along the inner curvatures of both the ascending and proximal descending arch. This may be a consequence of asymmetric forward momentum combined with the decline of net forward flow, and may contribute to diastolic filling of the coronary arteries [85,86].

Studies investigating normal pulmonary arterial flow revealed relatively uniformly distributed velocities across the cross section of the main pulmonary artery in early systole. During late systolic deceleration, a relatively high velocity central stream tends to migrate towards the outer curvature of the antero-superior wall [87,88]. A further change of direction into the right pulmonary artery is typically associated with right handed helical flow.

Recently, a number of studies have investigated derived parameters such as wall shear stress (WSS) and pulse wave velocity (PWV) in the healthy aorta. 4D velocity CMR was used to derive the normal distribution of vectorial WSS in the entire thoracic aorta. Marked regional variations of WSS found in these studies may help explain why atherosclerotic lesions tend to develop and progress at specific locations in the aorta such as the inner aortic curvature and supra-aortic branches [89]. The feasibility of aortic PWV estimation from 3D cine velocity data, exploiting its volumetric coverage, has been demonstrated, although temporal resolution remains suboptimal. The resulting PWV data reflecting global aortic compliance were in good agreement with previous 2D-PC studies [68].

### Blood Flow in Cardiovascular Disease

#### Heart

A number of studies have investigated abnormal intracardiac flow. Considerable changes in the presence, position and extent of vortices, as well as relative size of the flow components have been found in patients with dilated cardiomyopathy [75,76,90]. The vortices, which in the normal ventricle are constrained by proximity of the walls, have space to develop in a dilated ventricle and higher blood volume to conserve rotational momentum from one heart beat to the next. This can manifest either as a large rotating vortex if inflow is relatively tangential, or as a propagating ring vortex if inflow is more apically directed. In addition to the expected increase in *Residual Volume*, related to the high end systolic volume of the cavity, other flow components show changes such as a decrease in the *Direct Flow *[75,76].

**Figure 11:**
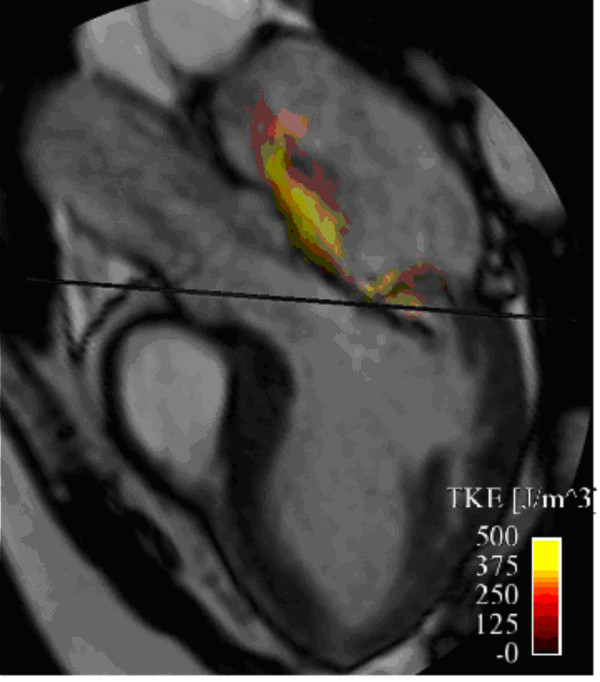
**Volume rendering visualization of turbulence intensity (turbulent kinetic energy, TKE) at mid-late systole in the left atrium of a patient with severe mitral regurgitation**. A better comprehension of the 3D spatial extension of the TKE can be obtained from additional file 8.

Multidirectional velocity acquisitions can be used for assessments of valve abnormalities before surgery and during follow-up [23]. It has been shown that 4D velocity CMR with retrospective valve-tracking can be used to accurately quantify net flow volumes through all four heart valves in patients with valvular regurgitation [74]. Findings demonstrated analogous average net flow volume through all four valves and good intra- and interobserver agreement for the assessment of regurgitation fraction [91]. Valvular stenosis and regurgitation, with associated high velocity jet flow, can modify intracardiac flow patterns. Elevated turbulent intensity values have been found in the heart at the break down of these jets in mild aortic and severe mitral regurgitation (see also figure 11 and additional file 8) [73,76]. Regurgitation through atrio-ventricular valves has been quantified using 4D velocity acquisition, potentially contributing to diagnosis and treatment planning in heart valve disease [91]. Vortices in the sinuses of Valsalva have been studied after different valve-sparing aortic root replacements [85,86] contributing to an ongoing discussion about the role of the sinuses and the importance of maintaining them in valve sparing surgical repair of aortic root ectasia. Blood flow patterns and turbulence intensity downstream from a prosthetic heart valve have been found to be dependent on the specific valve design [92]. Improved understanding of the hemodynamic consequences of these surgical procedures may contribute to surgical strategies that result in more physiological post operative flow.

#### Large vessels

A number of studies have described blood flow in patients with common vascular pathologies such as aortic atheroma, aneurysms, dissection, coarctation and graft repair, identifying regions of complex and potentially pathological flow [22,23,44,57,58,84].

A study using 4D velocity acquisition provided novel evidence for a potential source of otherwise cryptogenic embolic stroke. Pathline visualization showed a high incidence of retrograde flow connecting complex atheromatous plaques in the proximal descending aorta with inflow to the carotid arteries. It suggested that descending aortic atheroma, even if located several centimeters distal to the left common carotid artery, should be regarded as a potential source of embolic stroke [93].

**Figure 12:**
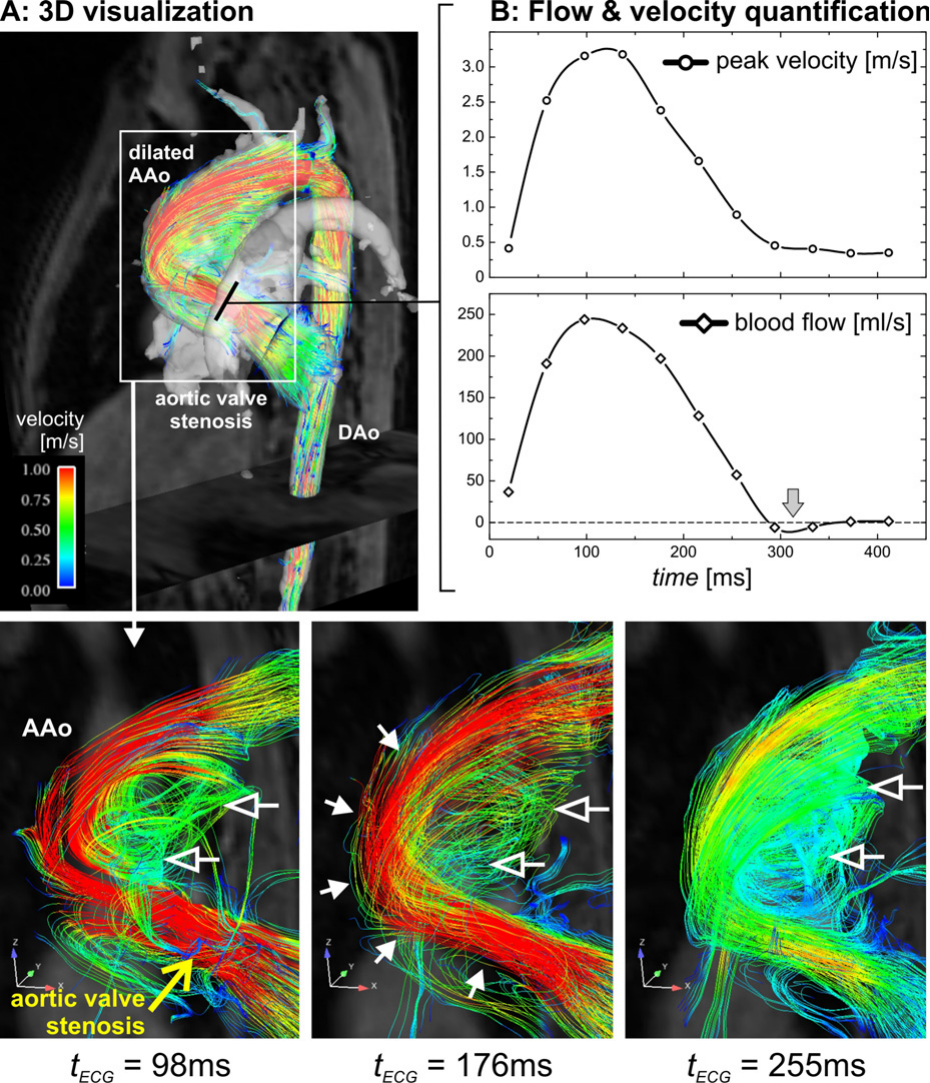
**3D flow visualization (A) and flow quantification (B) in a nine year old pediatric patient with aortic valve stenosis (aortic valve area = 1.2 cm**^**2**^**) and dilation of the ascending aorta (maximum diameter = 33 mm)**. Echocardiography demonstrated normal global cardiac function (ejection fraction EF = 72%) but substantial flow acceleration (peak velocity = 2.8 m/s) and an elevated pressure gradient (maximum pressure = 38 mmHg) at the level of the aortic valve. These findings were confirmed by retrospective quantitative analysis of the 4D PC-MRI data in an analysis plane above the aortic valve which revealed high systolic peak velocities (peak velocity = 3.2 m/s, max pressure gradient = 41 mmHg) but only very mild diastolic retrograde flow (gray arrow). 3D flow visualization using streamlines showed localized flow acceleration along the outer wall of the ascending aorta (solid white arrows) which developed into a vortex flow pattern (open arrows) occupying the shape of the aneurysm (AAo: ascending aorta, DAo: descending aorta).

Other studies showed that relatively subtle geometric changes of the ascending aorta, for example age related ectasia or moderate aortic valve stenosis, resulted in marked alteration of flow patterns in and downstream of the affected region [83,94]. In patients with aneurysms, abnormally pronounced helical (Figure 8) or vortical flow features were found (Figure 12). Reports on patients with bicuspid aortic valves (BAV) illustrated the link between valve geometry and patterns of flow. It was postulated that the eccentricity of jets and abnormal helical systolic flow in the ascending aorta of BAV patients might help identify patients at risk for development of ascending aortic aneurysm [58]. It has been shown that pulmonary hypertension coincides with the appearance of vorticity in the dilated main pulmonary artery [88].

Findings in a patient with aortic valve stenosis and dilated ascending aorta are illustrated in figure 12. The 4D velocity data were used to quantify regional blood flow and peak velocities in good agreement with results from echocardiography (for details see figure 12 legend). The abnormal flow patterns (accelerated flow channel along the outer ascending aortic wall and formation of a large recirculating vortex) in the aorta illustrate the effect of the pathological valve function on large scale flow.

Such studies can help to identify regions of abnormal turbulence and shear rates. From the literature it is known that unfavorable shear forces near the vessel wall can change endothelial function and create areas at risk for vascular remodeling [95]. Flow abnormalities may thus contribute to the development of cardiovascular disease such as aortic aneurysms or atherosclerosis. The derivation of secondary flow and vessel wall parameters from the 4D velocity data (wall shear stress, pulse wave velocity, pressure difference maps, etc.) may help to quantify flow alterations and identify new pathogenetic characteristics or risk factors.

Initial studies in larger cohorts of patients with aortic atherosclerosis have confirmed the expected increase of global pulse wave velocity (i.e. stiffening of the aorta) related to age and presence of atherosclerosis [68]. Wall shear stress analysis revealed that potentially atherogenic wall parameters (low wall shear stress and high oscillatory shear index) were located adjacent to the atheroma. In addition, preliminary results indicate that the predictive value of wall shear stress for plaque existence may depend on the aortic segment [96]. However, regional variations in the instability of flow, which can be associated with local peaks or fluctuations of wall shear stress, remain effectively invisible to the 4D visualization methods used. Moreover, the current spatial resolution of 4D velocity data is insufficient to resolve small scale boundary layers of arterial velocity profiles. WSS values should therefore be considered estimates of the shear rate of the blood near the vessel wall.

The more intense, turbulent instabilities associated with jet flow beyond a stenosis have been investigated, as outlined above, by turbulent intensity analysis [72,73]. Here abnormally rapid velocity fluctuations and gradients are associated with eddies and counter-eddies, generated in the para-jet shear layers and swept downstream. These dissipate kinetic energy as heat and results in a loss of pressure as well as large fluctuations in adjacent wall shear stress [97]. In figure 10, the velocity field at peak systole is visualized using streamlines while turbulence intensity is visualized by a volume rendering of the turbulent kinetic energy.

Studies in patients with aortic coarctation demonstrated that 4D velocity acquisition can help evaluate collateral blood flow as a potential measure of hemodynamic significance. Additionally, 3D visualization showed distorted flow patterns in the descending aorta after coarctation repair such as marked helical and vortical flow in regions of post-stenotic dilation [44].

#### Whole Heart and Large Vessels

By exploiting the trade-off that can be made between volume coverage and scan time, 3D flow maps covering the entire heart and surrounding large vessels can be acquired in a single acquisition. However, currently available techniques still require scan times of about 20-40 minutes for adequate spatial (~2-3 mm^3^) and temporal (40-50 ms) resolution.

**Figure 13:**
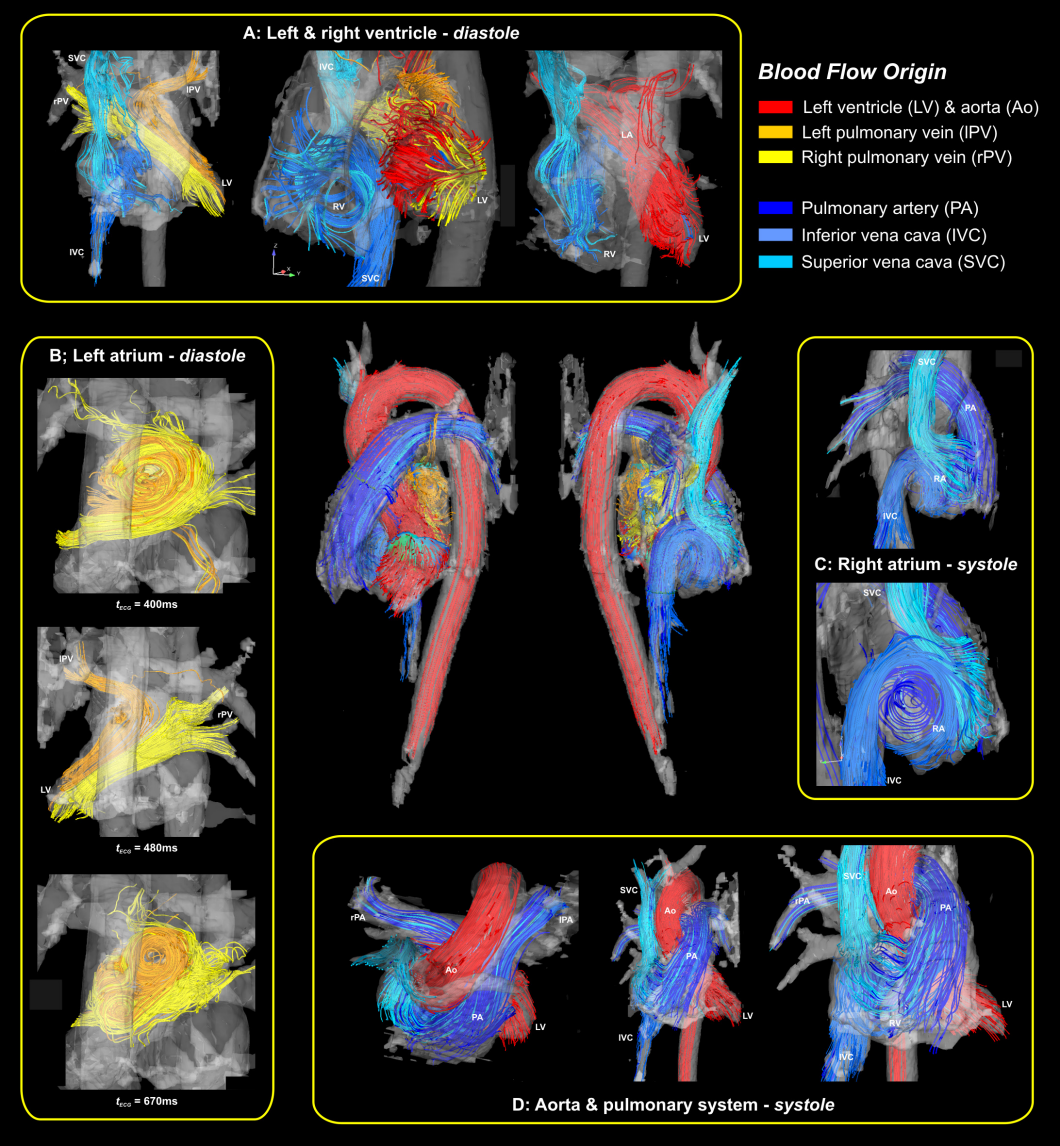
**Flow visualization of the heart and large vessels, viewed from several aspects, using streamlines to show the large scale intra-cavity flow structures in ventricular systole and diastole in a normal young volunteer**. Color coding is based on the streamline origin. Iso-surface representation of 3D PC-MRA data derived from the same data set was used to generate the semi-transparent outer cavity boundary.

Nevertheless, recent studies in normal subjects demonstrated that it is possible to characterize large scale flow through the entire heart and great vessels from a single free-breathing scan at a level of quantitative accuracy comparable to conventional 2D flow measurements [19,87]. The complete spatial and temporal coverage of the heart and surrounding vessels provides previously unattainable visualization of the changing, multidirectional flow fields as shown in figure 13. The representation of instantaneous velocity directions in the heart and surrounding vessels was achieved by 3D streamline calculation. Emitter planes were placed in the left and right pulmonary veins, the left ventricle, the ascending aorta, the inferior and superior vena cava, and the main pulmonary artery. To improve the visibility of flow paths, traces along the measured velocities were color coded according to their anatomic origin.

**Figure 14:**
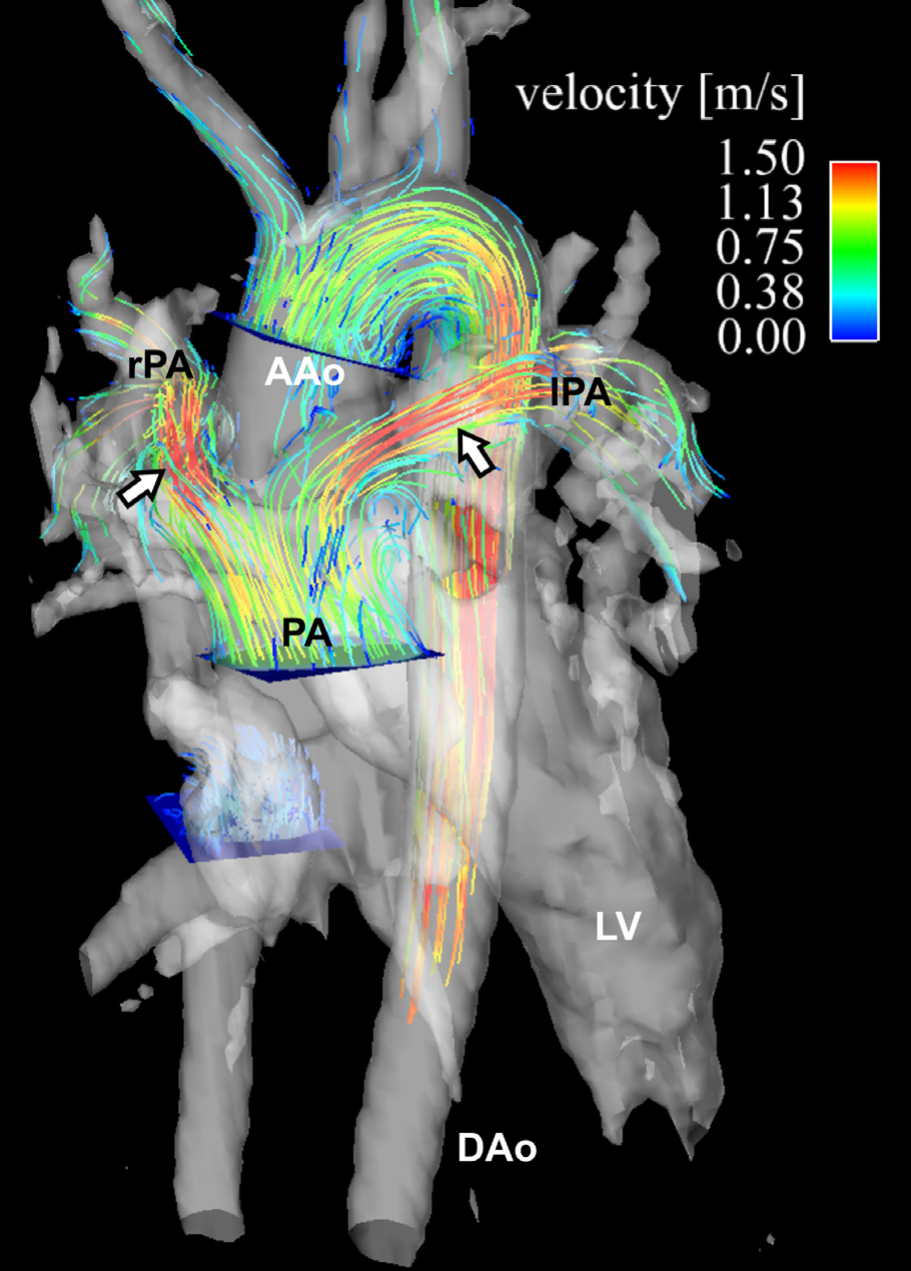
**3D cine velocity acquisition in a patient with transposition of the great arteries corrected by an arterial switch procedure showing the post-surgical course of the pulmonary arteries, straddling the aorta**. The 3D pathlines represent systolic flow from emitter planes in the ascending aorta (AAo) and main pulmonary artery (PA). Flow acceleration with peak velocities greater than 1.5 m/s in left (lPA) and right (rPA) pulmonary artery can be seen (white arrows) whereas the flow pattern in the aorta was normal. AAo: ascending aorta.

Such applications may be useful for evaluating abnormal flows in patients with more complex forms of congenital heart disease [19,98]. In these patients, the situation can be modified by surgical interventions. An example is shown in figure 14 showing flow paths after surgical correction of transposition of the great arteries. The altered cardiovascular geometry, with the pulmonary artery bifurcation straddling the aorta and flow acceleration in into the right and left branches can be appreciated.

## Discussion

4D velocity acquisitions provide a non-invasive method for the qualitative and quantitative characterization of blood flow in the heart and great vessels. A benefit compared to traditional 2D MR velocity mapping or Doppler ultrasound is provided full 3D coverage and the feasibility of retrospective analysis of flow at any location in the imaging volume. Visualization and quantification of cardiovascular flow and hemodynamic parameters such as wall shear rates or pressure gradients has improved and will likely continue to improve our understanding of normal and pathologically altered cardiovascular physiology.

For example, the retrograde streaming demonstrated in atheromatous descending aortas has brought to light a potential thrombo-embolic pathway in patients with otherwise cryptogenic stroke [93]. The technique may come to have roles in diagnosis and the planning of treatment. A number of studies have shown that relatively minor and unsuspicious alterations in cardiac and vascular anatomy such as a mild ascending aortic aneurysm or moderate valve disease resulted in surprisingly extensive alterations of flow. In addition, the ability to retrospectively quantify cardiovascular hemodynamics provides an advantage compared to standard diagnostic tools where often only qualitative measurements (e.g. valvular regurgitation) or simply anatomic dimensions (e.g. aneurysm diameter) are currently used. This points to a possible role for multidirectional flow velocity analysis rather than reliance on simple anatomical parameters in the evaluation and monitoring of treatment of cardiovascular disease with the goal of better risk stratify patients.

However, mainly because of the time and experience currently needed for appropriate acquisition and analysis, 4D acquisitions have yet to become accepted in routine clinical CMR practice as alternatives or adjuncts to more targeted combinations of breath-hold velocity map acquisitions. Echocardiography and Doppler ultrasound are still widely and routinely used for many of the indications targeted by 4D MR velocity mapping. Specifically in valvular disease where it is important to image the exact location of jet flow relative to the thin and mobile leaflets of valves echocardiography is currently more suitable than 4D velocity mapping. Nevertheless, 4D velocity acquisitions may be advantageous to measure flow through several valves and vessels in a single measurement, as may be needed in valve disease combined with a shunt lesion.

However, the diagnostic and predictive value of the flow visualizations and measurements need further investigation. Larger trials, including studies before and after therapy or during the progression of disease will be needed to evaluate the clinical usefulness 4D velocity acquisition.

It should be noted that similar 4D velocity mapping techniques have been applied in different vascular territories such as peripheral vessels [99], carotid arteries [25,100], large intra-cranial arteries [27,28,101,102], and to the velocities of myocardial movements and deformation [32-34]. 4D velocity acquisitions can be used independent from the used MRI platform without major differences with respect to possible implementations of the MR pulse sequence.

### Limitations

A key limitation is the time taken to acquire and analyse 4D velocity datasets. The acquisition times may be too long for the tolerance of some patients, and irregular heart rate or breathing patterns tend to result in suboptimal data. Towards reduction of acquisition times, new spatio-temporal imaging acceleration techniques (k-t BLAST, k-t GRAPPA, etc.) are promising since redundancies in two spatial encoding and the temporal dimensions can be utilized to speed up data acquisition [103,104]. It should be noted that a limitation of such techniques when applied to aortic flow acquisition may be related to temporal blurring which can lead to inaccuracy in flow acquisitions [105].

New methods based on the combination of phase contrast MRI and fast sampling strategies, e.g. echo-planar imaging and radial imaging with 3D PC-VIPR, have been reported and are promising for further reduction in total scan time and/or increased spatial or temporal resolution [20,87]. A number of studies have already demonstrated the potential of radial imaging techniques for the assessment of vascular function with increased efficiency compared to conventional methods [20,53,106-108]. Particularly whole heart 4D velocity acquisitions with their long scan times which severely limit their clinical feasibility could benefit from new sampling and parallel imaging strategies. In this context, 4D velocity mapping has potential to benefit from imaging at higher field strength. Due to the small flip angles used radio frequency power deposition does not pose a major problem. The gain in signal-to-noise ratio (SNR) associated with high field CMR can be used for improved image quality and translates to reduced noise in the velocity encoded images. Recently reported results indicate a considerable gain in SNR, which may be used to increase spatial and/or temporal resolution or reduce the noise associated with parallel imaging [109].

Imaging at even higher magnetic field strength (7T) may thus be promising for achieving high spatial resolution and assessing small vascular structures (e.g. second and third branches of intra-cranial arteries) which are difficult to assess with currently existing 4D velocity implementations. One of the ultimate goals, the acquisition of time-resolved 3D hemodynamics in the coronary arteries, has to date not been accomplished. Due to their low diameter, complex structure, and particular substantial motion during the cardiac cycle, the assessment of coronary 4D velocities is expected to be challenging even at ultra-high fields. We speculate that a combination of spatio-temporal image acceleration, improved and reliable respiration synchronization, high field, and possibly even intra vascular contrast agents may be necessary.

The time needed for acquisition also means that, while relatively large scale cyclically repeating flows can be visualized, smaller scale flow instabilities are not. In the heart and large vessels, in some regions more than others, blood flow tends to fragment and mix through fluctuating eddy formation, particularly in regions of flow separation and high shear. These are inevitable given in the velocities, viscosity, dimensions, diameter-changes and direction-changes of normal as well as abnormal cardiovascular flow fields. Flow instabilities may be glimpsed in vivo as local variations of blood signal in breath-hold or real-time CMR cine images, particularly when flow in a dilated cavity is unusually slow. However, CMR lacks the spatio-temporal resolution to show all instabilities. They are generally too small and rapidly passing to be resolved, particularly by prolonged 4D velocity acquisitions. This has implications for the interpretation of some attempted measurements, notably those of wall shear stress. Powerful small scale velocity fluctuations, as present in disturbed or turbulent blood flow, can however be displayed from a 4D PC-MRI acquisition by quantification of the intravoxel standard deviation [72].

In addition to long acquisition times, another drawback of 4D velocity CMR is related to the complex and often time-consuming post acquisition data analysis. More automated methods for flow visualization and retrospective quantification would thus be helpful for applications within a clinical workflow. New software tools and algorithms need to be developed, for example to define standardized analysis planes in routinely acquired 4D velocity data. Similarly, for all aorta or cardiac studies, standard flow images might be generated automatically to expedite interpretation of the data sets.

The spatial resolution of 4D acquisitions is likely to be suboptimal for small vessel regions and jet flows, and this affects e.g. the accuracy of vascular segmentation by 3D phase contrast angiography from the same acquisition. The moving boundaries of the heart cavities and atrio-ventricular valves are relatively hard to determine by the approach. Precisely registered cine acquisitions could help, although this may depend on the use of a corresponding respiratory position.

## Conclusion

Comprehensive 4D velocity CMR imaging, visualization strategies, and quantification of cardiovascular hemodynamics are contributing to the understanding of cardiac and vascular pathologies, but inherent limitations of the technique should be born in mind. Due to the time currently needed to acquire and analyse the datasets, they have yet to become accepted in routine clinical CMR practice as alternatives or adjuncts to targeted 2D breath hold velocity measurements of flow.

## Competing interests

The authors declare that they have no competing interests.

## Authors' contributions

MM was responsible for studies and presented data regarding aortic and whole heart 4D velocity mapping and quantitative analysis of flow wall parameters, TE was responsible for studies and presented data regarding cardiac and aortic 4D velocity mapping and quantitative analysis of flow and turbulent kinetic energy, MM and TE drafted the manuscript, PK participated in critical revision of the manuscript and review of the current state and limitations of 4D MR velocity mapping. All authors participated in the literature review and read and approved the final manuscript.

## Supplementary Material

Additional file 1 Time-resolved velocity vector field and planar color coding in a plane longitudinally transecting the thoracic aorta in a normal volunteer. Click here for file

Additional file 2 Time-resolved 3D pathlines in a normal thoracic aorta illustrating the temporal evolution of blood flow during the cardiac cycle. Click here for file

Additional file 3 **Time-resolved 3D pathline visualization of blood flow in the heart and the great vessels**. Pathlines are emitted from planes located at the mitral valve (red-yellow) and the tricuspid valve (blue-turquoise) at early and late diastolic ventricular inflow. A separately acquired balanced steady-state free precession three-chamber image provides anatomical orientation. Click here for file

Additional file 4 **Rotating view of flow characterization in the heart of a healthy volunteer at late diastole**. Vortex cores are shown as white isosurfaces. In addition, short streamlines have been emitted from these isosurfaces. (Partial) vortex rings can be seen below the mitral valve and the tricuspid valve. Click here for file

Additional file 5 Time-resolved 3D pathlines in the aorta of a patient with and ascending aortic aneurysm illustrating the dynamics of disturbed blood flow during the cardiac cycle. Click here for file

Additional file 6 **Time-resolved pathline visualization of all blood flow involved in one cardiac cycle in the left ventricle (LV) of a healthy, 61 year old male**. The pathlines are color coded according to their behavior over the cardiac cycle in 4 different components: *Direct Flow *(green) enters the LV during diastole and leaves the LV during systole in the analyzed heart beat, *Retained Inflow *(yellow) enters the LV during diastole but does not leave during systole in the analyzed heart beat, *Delayed Ejection Flow *(blue) starts and resides inside the LV during diastole and leaves during systole, *Residual Volume *(red) resides in the LV during more than two cardiac cycles[43]. Click here for file

Additional file 7 **Time-resolved volume rendering of turbulence intensity (turbulent kinetic energy, TKE) in a patient with an aortic coarctation located distal to the left subclavian artery**. The elevated values of turbulent kinetic energy in the ascending aorta were caused by a minimally obstructive subvalvular membrane, exemplifying the sensitivity of this measure to minor abnormalities in blood flow. A semi-transparent three-dimensional isosurface rendering of CE-MRA data is shown for orientation. Click here for file

Additional file 8 Time-resolved volume rendering visualization of turbulence intensity (turbulent kinetic energy, TKE) in the left atrium of a patient with severe mitral regurgitation. Click here for file
